# Baroreflex sensitivity in facioscapulohumeral muscular dystrophy

**DOI:** 10.14814/phy2.15277

**Published:** 2022-04-21

**Authors:** Miguel Anselmo, Shandon Coffman, Mia Larson, Kathryn Vera, Emma Lee, Mary McConville, Michael Kyba, Manda L. Keller‐Ross

**Affiliations:** ^1^ 5635 Division of Physical Therapy Medical School University of Minnesota Minneapolis Minnesota USA; ^2^ Sidney Kimmel Medical College Thomas Jefferson University Philadelphia Pennsylvania USA; ^3^ Health and Human Performance Department University of Wisconsin–River Falls River Falls Wisconsin USA; ^4^ College of Saint Benedict St. Joseph Minnesota USA; ^5^ 5635 Department of Pediatrics and Lillehei Heart Institute University of Minnesota Minneapolis Minnesota USA; ^6^ 5635 Division of Rehabilitation Science Medical School University of Minnesota Minneapolis Minnesota USA

**Keywords:** autonomic function, blood pressure, FSHD, heart rate variability, muscular dystrophy

## Abstract

Facioscapulohumeral muscular dystrophy (FSHD), a common form of muscular dystrophy, is caused by a genetic mutation that alters DUX4 gene expression. This mutation contributes to significant skeletal muscle loss. Although it is suggested that cardiac muscle may be spared, people with FSHD have demonstrated autonomic dysregulation. It is unknown if baroreflex function, an important regulator of blood pressure (BP), is impaired in people with FSHD. We examined if baroreflex sensitivity (BRS) is blunted in patients with FSHD. Thirty minutes of resting BP, heart rate, and cardiovagal BRS were measured in 13 patients with FSHD (age: 50 ± 13 years, avg ± SD) and 17 sex‐ and age‐matched controls (age: 47 ± 14 years, *p* > 0.05). People with FSHD were less active (Activity Metabolic Index, AMI) (FSHD: 24 ± 30; controls: 222 ± 175 kcal/day; *p* < 0.001) but had a similar body mass index compared with controls (FSHD: 27 ± 4; controls: 27 ± 4 kg/m^2^; *p* > 0.05). BRSup (hypertensive response), BRSdown (hypotensive response), and total BRS were similar between groups (BRSup: FSHD: 12 ± 8; controls: 12 ± 5 ms/mmHg; BRSdown: FSHD: 10 ± 4; controls: 13 ± 6 ms/mmHg; BRS: FSHD: 14 ± 9; controls: 13 ± 6 ms/mmHg; *p* > 0.05). Mean arterial pressure was similar between groups (FSHD: 96 ± 7; controls: 91 ± 6mmHg). Individuals with FSHD had an elevated heart rate compared with controls (FSHD: 65 ± 8; controls: 59 ± 8 BPM; *p* = 0.03), but when co‐varied for AMI, this relationship disappeared (*p* = 0.39). These findings suggest that BRS is not attenuated in people with FSHD, but an elevated heart rate may be due to low physical activity levels, a potential consequence of limited mobility.

## Graphical abstract

The current study provides evidence that baroreflex and heart rate variability in individuals with FSHD are preserved, despite their lower physical ability indicated by the FSHD‐health index. Further, our observations suggest that an elevated heart rate in FSHD may be indicative of physical inactivity or deconditioning, which may put this group at risk for future CVD or cardiac events, but this appears to be secondary to FSHD or overexpression of the DUX4 gene mutation, as we did not observe alterations in autonomic function in this group.

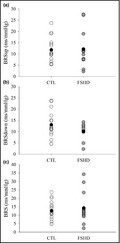

## 1 INTRODUCTION

Facioscapulohumeral muscular dystrophy (FSHD) is the third most common type of muscular dystrophy, present in about 1/20,000 of the population (Mavrogeni et al., 2018). It is caused by the reactivation of the double homeobox protein 4 (DUX4) in skeletal muscle, which is silenced after early embryonic development in most organs (Lim et al., 2020). The expression of the DUX4 gene influences many complex pathways, eventually leading to skeletal muscle cell death (Bosnakovski et al., 2008; Geng et al., 2012). Thus, individuals with FSHD exhibit progressive skeletal muscle atrophy and functional declines in strength and movement (Emmrich et al., 2005; Wang & Tawil, 2016) which present first in the face, shoulder girdle, and upper‐arm regions, and then progress to the lower extremities (Statland & Tawil, 2016; Wang & Tawil, 2016).

Although not observed by all (Tawil et al., 2015; Trevisan et al., 2006), several studies have noted cardiac and autonomic dysfunction in people with FSHD (Berlit & Stegaru‐Hellring, 1991; Finsterer et al., 2005; Laforêt et al., 1998; Stevenson et al., 1990). For example, electrocardiogram abnormalities (Berlit & Stegaru‐Hellring, 1991; Finsterer et al., 2005; Laforêt et al., 1998; Stevenson et al., 1990), abnormal ventricular hypertrophy (Finsterer et al., 2005), and cardiomyopathy (Laforêt et al., 1998) were amongst the cardiac symptoms exhibited by patients with FSHD. Both cardiac and autonomic dysfunctions are of clinical relevance and linked to hypertension (Lucini et al., 2002), risk of future cardiovascular events (Ormezzano et al., 2008), and heart failure (La Rovere et al., 2001; Thames et al., 1993). It is unclear, however, whether the dysfunction noted in these studies was a result of the genetic mutation or of deconditioning, a consequence of the physical disability that manifests from FSHD.

Previous work has demonstrated that individuals with FSHD exhibit exercise intolerance (Vera et al., 2022), driven by loss of skeletal muscle (Preston et al., 1999); are at higher risk for developing sarcopenic obesity (Vera et al., 2020); and are usually less physically active than people without FSHD as a result of their disability (Vera et al., 2020, 2021). This decrease in activity leads to physical deconditioning, which is known to contribute to the acceleration of cardiovascular disease (CVD) development through attenuated autonomic function (Coupé et al., 2009; Maher et al., 2017). The potential increased risk of CVD associated with autonomic dysfunction through low levels of cardiorespiratory fitness could suggest a higher risk of CVD in adults with FSHD.

The baroreflex, a critical regulatory component of the autonomic nervous system (ANS), is a neurally mediated, negative feedback mechanism that is an important contributor to blood pressure (BP) homeostasis. Earlier studies demonstrated that arterial baroreflex sensitivity (BRS) primarily contributes to the short‐term regulation of BP (Lanfranchi & Somers, 2002), while others suggest a strong contribution to long‐term BP regulation (Lohmeier, 2001; Lohmeier et al., 2004). Baroreflex sensitivity has been observed to be low in physically deconditioned individuals (Hughson & Shoemaker, 2015), as well as in hypertensive patients at high risk of developing CVD (Gordin et al., 2016). Whether BRS is attenuated in adults with FSHD, however, remains unknown. Understanding BRS in adults with FSHD is a critical step in characterizing the CVD risk that may be associated with FSHD, thereby facilitating early interventions and treatment strategies to improve cardiovascular health and a healthy lifespan in people with FSHD. Thus, the objective of the present study was to examine if BRS is impaired in patients with FSHD. We hypothesized that BRS would be attenuated in the FSHD group as compared with controls.

## 2 METHODS

### 2.1 Participants

Thirteen adults with FSHD (age 50 ± 13 years; four females, nine males; one Hispanic, twelve European American) and seventeen healthy age‐ and sex‐matched individuals (age 47 ± 14 years; five females, twelve males; one of African descent, two of Asian descent, one Hispanic and thirteen European Americans) were recruited to participate in this study. Inclusion criteria consisted of: ≥18 years of age and no prior history of cardiovascular, pulmonary, orthopedic, or neuromuscular disorders other than FSHD; female participants were excluded if they were currently pregnant or breastfeeding (Dewey, 1997; Lof et al., 2005). Menstrual cycle was not controlled for in the female participants. This study is part of a larger clinical study (Vera et al., 2021). Participants provided written informed consent prior to any procedures being performed. The study was approved by the University of Minnesota Institutional Review Board and conducted in accordance with the Declaration of Helsinki.

### 2.2 Study design

Participants completed one study visit at which they completed a general medical questionnaire to rule out cardiovascular, neuromuscular, pulmonary or orthopedic disorders. They also completed a physical activity questionnaire to calculate their activity metabolic index score (AMI) (Richardson et al., 1994). To determine the functional ability level and severity of FSHD symptoms, people with FSHD completed a Facioscapulohumeral Muscular Dystrophy Health Index questionnaire (FSHD‐HI) (Johnson et al., 2012). These surveys were scored on a scale of 0 to 100, where 100 reflects the worst subjective level of disease severity. Females were required to provide a urine sample prior to study procedures to ensure they were not pregnant. Participants fasted and had no caffeine for at least 12 h prior to the study.

### 2.3 Procedures

After written informed consent, height and weight were obtained, participants rested in a supine position and were instrumented with non‐invasive devices to measure heart rate (HR) and BP. Data was collected over 30 min of rest.

Heart rate was measured with a three‐lead electrocardiogram (ECG) (ADInstruments), and BP was recorded via a small cuff placed on the annular or middle finger of the participant's non‐dominant hand (Human non‐invasive blood pressure [NIBP], ADInstruments). Manual brachial BP was obtained to calibrate the NIBP system. Heart rate variability and BRS were calculated from the ECG as well as the ECG and BP measurements, respectively.

### 2.4 Data analysis

Data was sampled and recorded at 1000 samples/s by a high‐performance data acquisition device (PowerLab, ADInstruments). Cardiovascular variables were recorded continuously throughout the 30‐min rest period. Spontaneous cardiovagal BRS was quantified using the sequence method (Bertinieri et al., 1985). The sequence method identifies three or more consecutive beats where systolic BP (SBP) and R‐R interval (RRI) concurrently increase or decrease by at least 1 mmHg or 4 ms, respectively (Wessel et al., 2020). Sequences of three or more beats changing in the same direction were only included in the calculation if the correlation coefficient was greater than 0.7 (i.e., *r* > 0.7) (Parati et al., 2000; Smyth et al., 1969). Sequences were manually inspected to exclude artifacts. Baroreflex sensitivity to hypertensive stimuli (BRSup) and hypotensive stimuli (BRSdown) were quantified, as they have been reported to correlate with the phenylephrine pressor (hypertensive stimuli) stimuli and nitroprusside depressor (hypotensive) baroreflex test sensitivities, respectively (Rudas et al., 1999; Watkins et al., 1995). Thus, BRSup is the average of the slopes where SBP and RRI increase simultaneously, and BRSdown is the average of the slopes where SBP and RRI decrease simultaneously.

Heart rate variability was calculated with LabChart software (ADInstruments). Heart rate variability was reported as the average RRI, standard deviation of normal‐normal intervals (SDNN), and root mean square of successive differences between normal heartbeats (RMSSD) for time‐domain analyses, and as high frequency (HF) power for the frequency domain analysis. The SDNN is the standard deviation of intervals between heartbeats with ectopic beats removed (i.e., normal intervals), while RMSSD is the root mean square of successive normal intervals. High frequency power is a reliable estimation of parasympathetic activity and ranges between frequencies of 0.15–0.4 Hz (Pomeranz et al., 1985). The low frequency (LF) domain, although controversial, represents a combination of parasympathetic activity, sympathetic activity and baroreflex function (Randall et al., 1991). Thus, because of the non‐specificity of LF power, this variable, along with LF/HF power, were not included in the analyses.

### 2.5 Statistical analysis

Data was evaluated for normality using the Shapiro‐Wilk test prior to analysis. Data that was not normally distributed was subsequently analyzed with nonparametric methods. Demographic characteristics of the participants were analyzed and reported as means and standard deviations. Two‐sample independent *t*‐tests were used to compare age, body mass index (BMI), HR, SBP, mean arterial pressure (MAP), RMSSD, RRI, SDNN, and BRSdown between FSHD and CTL. The Mann‐Whitney U‐test was used to compare activity metabolic index (AMI), diastolic blood pressure (DBP), HF power, BRSup, and total BRS between these groups. To determine if BMI, AMI and use of antihypertensive or antidepressant medication influenced autonomic and cardiovascular variables, analyses were repeated including these factors as covariates.

For the HRV analyses (i.e., RMSSD, RRI, SDNN, and HF power), data from one individual with FSHD was excluded due to an arrhythmia. In all analyses, outliers were identified using the interquartile rule with a multiplier of 2.2 (Hoaglin et al., 1986). Following identification of outliers, one additional individual with FSHD was excluded from HF power analysis, and three individuals (all FSHD participants) were excluded from BRSdown analysis. Thus, the final participant numbers were *n* = 17 for CTL and *n* = 12 for FSHD for RMSSD, RRI, and SDNN; *n* = 17 for CTL and *n* = 11 for FSHD for HF power; and *n* = 17 for CTL and *n* = 10 for FSHD for BRSdown. In all other analyses, *n* = 17 for CTL and *n* = 13 for FSHD. To evaluate effect size, Cohen's *d* was calculated for hemodynamic and HRV variables. For all statistical analyses, SPSS v27.0 (IBM) was used. The ⍺ level for significance was set at 0.05.

## 3 RESULTS

Control and FSHD participants were similar in age, BMI, and the use of anti‐hypertensive and antidepressant medications (*p* > 0.05), but AMI was lower in the FSHD group (*p* < 0.001, Table 1).

**TABLE 1:** Participant characteristics and baseline cardiovascular data

	FSHD	Control
Female (n)	4	5
Male (n)	9	12
Age (range, years)	50 ± 13 (31–72)	47 ± 14 (25–75)
BMI (kg/m^2^)	27 ± 4	27 ± 4
AMI (kcal/day)	24 ± 30	222 ± 175*
FSHD‐HI (au)	29 ± 13	—
Anti‐hypertensive medications (n)	1	2
Anti‐depressant medications (n)	3	4
Heart rate (bpm)	65 ± 8	59 ± 8*
Systolic BP (mmHg)	124 ± 11	122 ± 11
Diastolic BP (mmHg)	80 ± 8	76 ± 8
Mean arterial BP (mmHg)	96 ± 7	91 ± 6

Note

Control and FSHD participants were similar in age, BMI, and the use of medications (*p* > 0.05). Activity metabolic index (AMI) was lower in the FSHD group (*p* < 0.001) and heart rate (beats/min, bpm) was greater in the FSHD group (*p* = 0.03). Data is presented as mean ± SD.

Abbreviations: au, arbitrary units; BMI, body mass index; BP, blood pressure; FSHD‐HI, Facioscapulohumeral muscular dystrophy health index.

* Significantly different than controls, *p* < 0.001.

**FIGURE 1:**
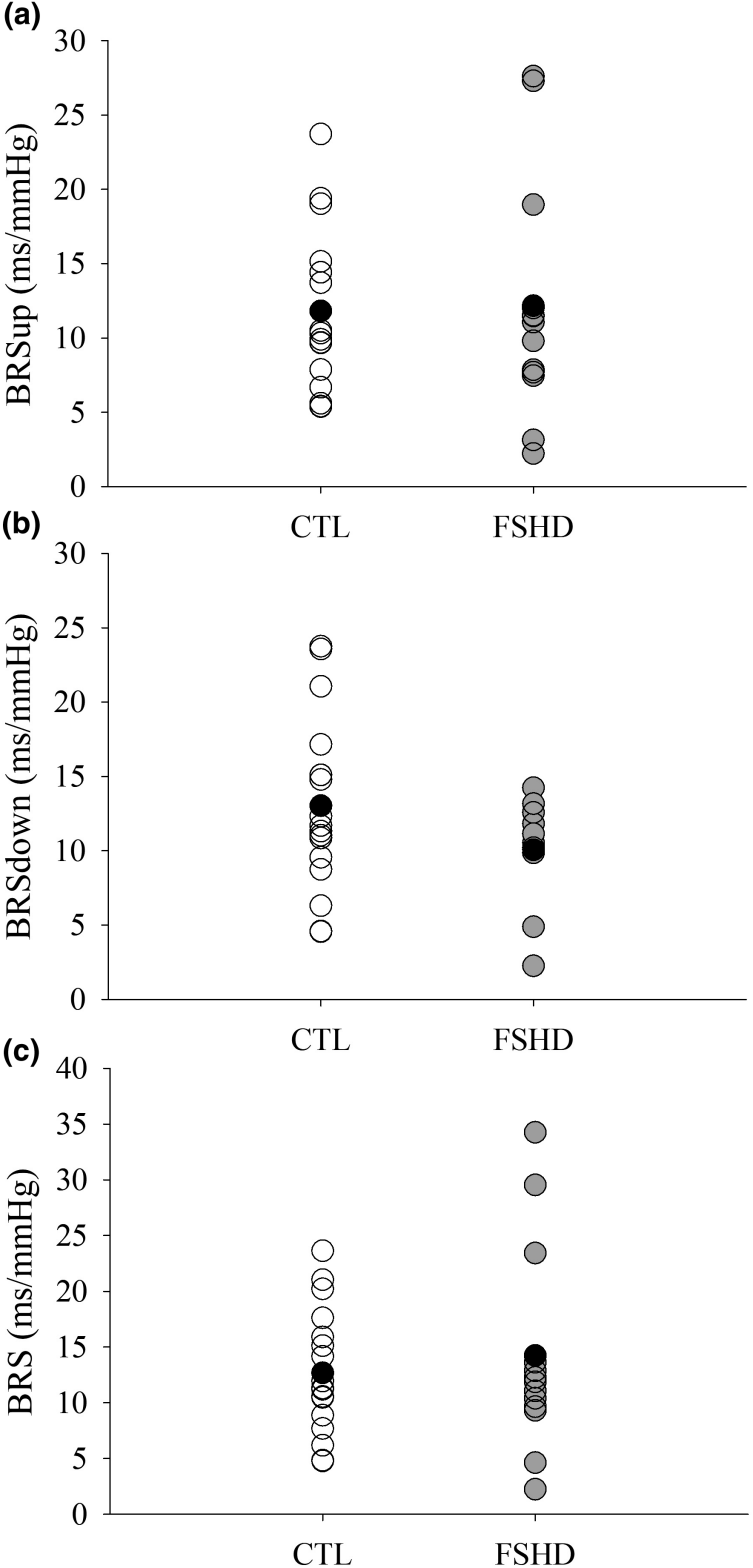
Cardiovagal baroreflex sensitivity. (a) BRSup: “up” cardiovagal baroreflex sensitivity, (b) BRSdown: “down” cardiovagal baroreflex sensitivity, (c) Total BRS. BRS measures were similar between controls and FSHD (*p* > 0.05). Black circles indicate the mean value for each group

**FIGURE 2:**
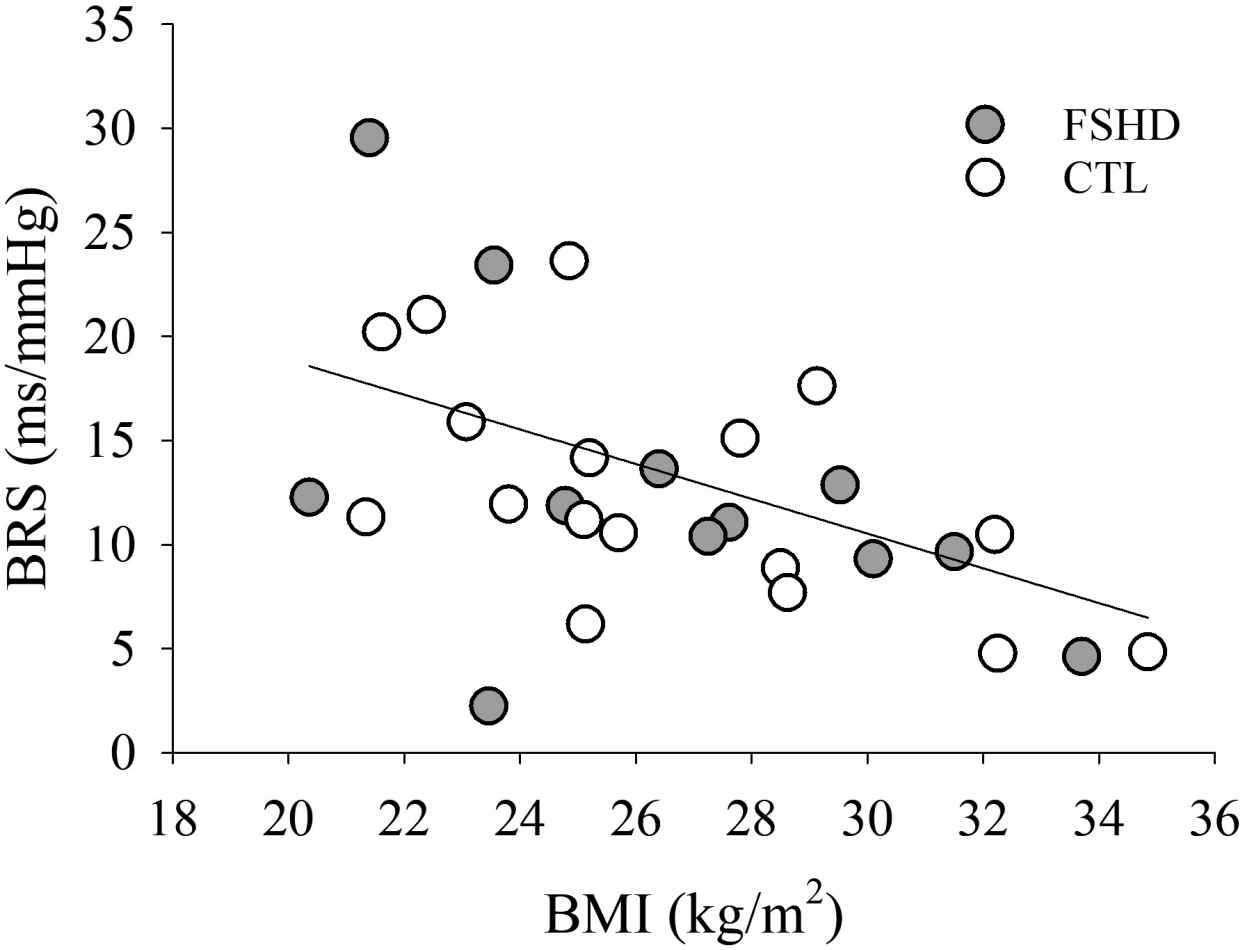
Relationship between body mass index (BMI) and baroreflex sensitivity (BRS) (*r* = −0.56, *p* < 0.001)

**FIGURE 3:**
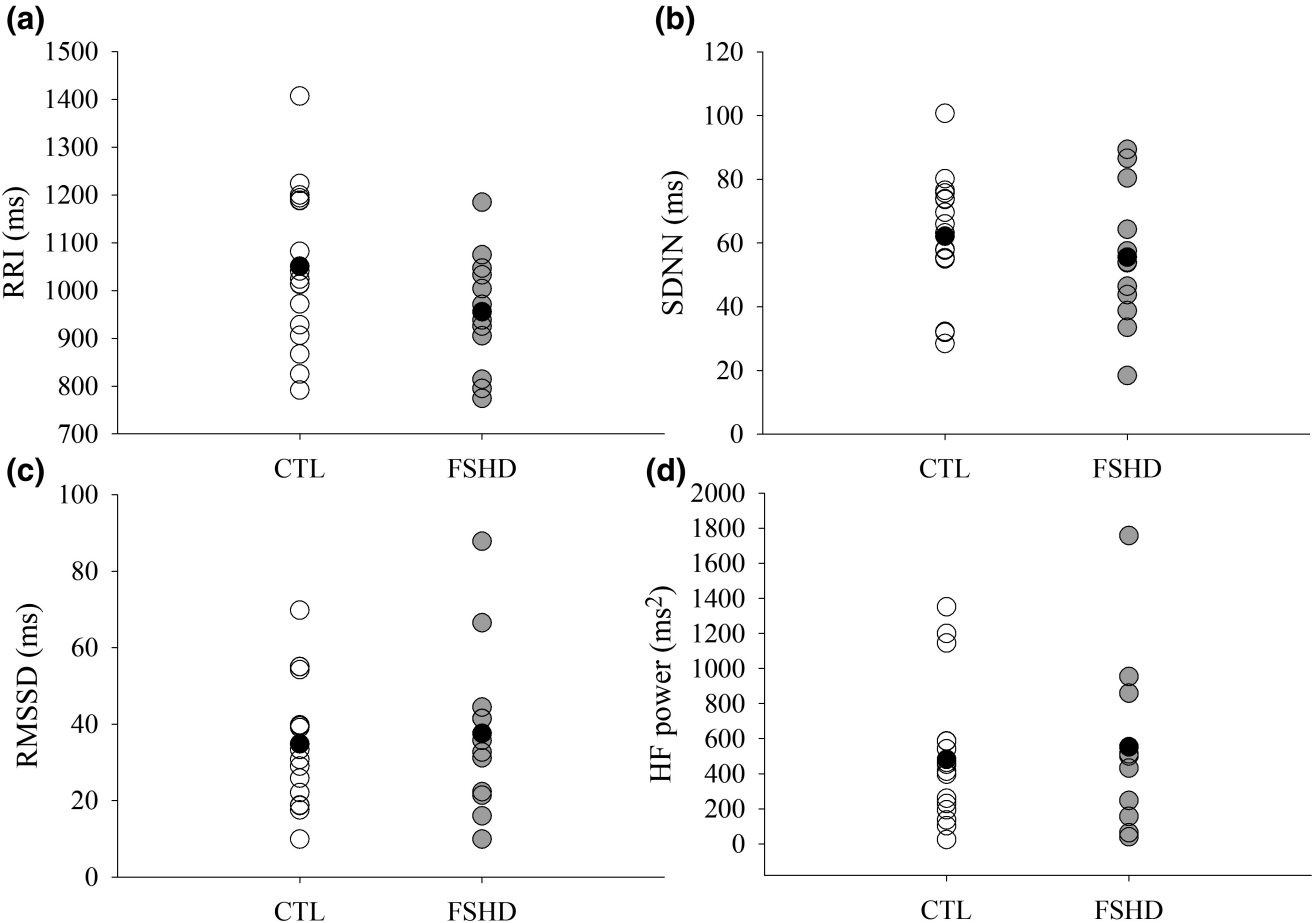
(a) R‐R interval (RRI) (FSHD, *n* = 12), (b) standard deviation of N‐N intervals (SDNN), (FSHD, *n* = 12), (c) root mean square of successive differences between normal heartbeats, (RMSSD) (FSHD *n* = 12), (d) high frequency power (HF), (FSHD, *n* = 11). CTL, control; FSHD, facioscapulohumeral dystrophy. HRV measures were similar between controls and FSHD (*p* > 0.05). Black circles indicate the mean value for each group

Neither BRSup (*d* = 0.05, *p* = 0.9, Figure 1a), BRSdown (*d* = 0.57, *p* = 0.17, Figure 1b), nor total BRS (*d* = 0.21, *p* = 1.0, Figure 1c) differed between FSHD and CTL groups. In addition, an inverse relationship was observed between BMI and BRS when groups were combined (*r* = −0.56, *p* < 0.001, Figure 2). However, BRS was not associated with AMI (*r* = 0.06, *p* = 0.77). Similarly, HRV measures were similar between groups (RRI: *d* = 0.64, *p* = 0.10, Figure 3a; SDNN: *d* = 0.02, *p* = 0.39, Figure 3b; RMSSD: *d* = 0.15, *p* = 0.70, Figure 3c; HF: *d* = 0.16, *p* = 0.78, Figure 3d). Body mass index was also inversely associated with RMSSD (*r* = −0.44, *p* = 0.02) and trended to be associated with HF power (*r* = −0.37, *p* = 0.06) when groups were combined. In addition, no associations were observed when groups were separated (*p* > 0.05).

Heart rate and BP data are located in Table 1. Heart rate was significantly higher in the FSHD group than in the CTL group (*d* = 0.09, *p* = 0.02, but when co‐varying for AMI, HR was similar between groups (*p* = 0.38). In addition, there was a trend for HR to be inversely associated with AMI, indicating that individuals with elevated HR were less physically active (*r* = −0.34, *p* = 0.08). Systolic BP (*d* = 0.22, *p* = 0.56), DBP (*d* = 0.59, *p* = 0.86), and MAP (*d* = 0.68, *p* = 0.08) were slightly but not significantly elevated in FSHD. The FSHD‐HI score was not correlated with any cardiovascular or autonomic measures (*p* > 0.05).

## 4 DISCUSSION

Although previous literature suggests individuals with FSHD demonstrate autonomic dysfunction, novel findings from this study reveal that BRS and HRV were similar in people with FSHD compared with controls. We did, however, observe an elevated HR in people with FSHD compared with controls that was mitigated when co‐varying for physical activity levels. Further, regardless of physical activity or disease state, we observed a strong relationship between BMI, baroreflex function and HRV, indicating that individuals with a lower BMI have a greater ability to modulate blood pressure and demonstrate greater parasympathetic activity, respectively.

### 4.1 Cardiovascular function and baroreflex sensitivity in adults with FSHD

Cardiac abnormalities have been observed in animal models and humans with FSHD. This is the first study, however, to investigate if BRS is altered in adults with FSHD. Indeed, studies investigating a relationship between any type of muscular dystrophy and BRS are sparse. In a mouse model of Duchenne muscular dystrophy, Chu et al. (2002). observed an attenuated BRS, while Sabharwal et al. (2015) reported that autonomic dysfunction preceded pathological cardiac hypertrophy in their mouse model of muscular dystrophy. Cardiac abnormalities in adults with FSHD that have been observed include left ventricular myocardial thickening (Finsterer et al., 2005) and abnormal ECG readings in the form of biatrial P‐waves, impaired sinoatrial node and atrioventricular node conduction, as well as atrial flutter or fibrillation (Laforêt et al., 1998; Stevenson et al., 1990). In addition, Ducharme‐Smith et al. demonstrated that in 104 individuals with genetically confirmed FSHD, the most common cardiac abnormalities were a right bundle branch block and mitral valve prolapse (Ducharme‐Smith et al., 2021).

Although several studies report cardiac and autonomic involvement in patients with FSHD (Berlit & Stegaru‐Hellring, 1991; Finsterer et al., 2005; Laforêt et al., 1998; Stevenson et al., 1990), no relationship with disease severity has been observed. The lone study to observe an association between disease severity and cardiac implications was conducted by Berlit and colleagues, where 20 patients with several types of muscular dystrophy were observed to have ECG abnormalities and hypertrophic cardiomyopathy (Berlit & Stegaru‐Hellring, 1991). The authors concluded that patients with progressive muscular dystrophy are more likely to exhibit cardiac symptoms as compared to those with stable muscular dystrophy. Collectively, with the majority studies demonstrating cardiac dysfunction, one might expect an attenuated BRS in FSHD, which would suggest impaired ability to modulate BP. Our findings of intact autonomic function in FSHD were surprising given that cardiac dysfunction can contribute to (Martinka et al. 2005) or precede baroreflex dysfunction (Sabharwal et al., 2015).

In the current study, BRStotal was slightly, but not significantly, elevated in FSHD compared with CTL, but when partitioned into BRSup and BRSdown, only BRSup was minimally elevated in FSHD than CTL. BRSdown was slightly depressed, albeit not significantly, in FSHD compared with CTL. The effect size was moderate, indicating the potential for the baroreflex to have a reduced ability to increase BP from an acute decline (Rudas et al., 1999) in FSHD. However, it is important to distinguish between the clinical significance of BRSup and BRSdown. BRSdown generally provides less insight into HTN and CVD risk than BRSup, as BRSup indicates the ability to decrease BP when it acutely increases. Further, BRSup, but not BRSdown, was found to be a significant predictor of the propensity for developing fatal arrhythmias after a heart attack in dogs 3–4 weeks after a myocardial infarction (Billman et al., 1982). Thus, an attenuated BRSup may be linked to the future development of fatal arrhythmias, whereas a small impairment in BRSdown may not be clinically meaningful. Regardless, there was not a significant impairment in either BRSup or BRSdown in adults with FSHD. Our observations of a relationship between BRS and BMI when groups are combined, coupled with the lack of association between FSHD‐HI and autonomic function measures, could suggest that autonomic dysregulation may be more strongly linked to body composition than to having FSHD or to the level of disability experienced by people with FSHD.

### 4.2 Heart rate variability in adults with FSHD

Altered HRV parameters are associated with increased risk of CVD and sudden cardiac events (Cygankiewicz & Zareba, 2013). Previous studies suggest an attenuated HRV in adults with FSHD (Della Marca et al., 2010). For example, Della Marca et al. observed that a patient's clinical severity of FSHD was inversely correlated with HF power and positively associated with LF power (Della Marca et al., 2010). These authors suggest that their findings indicate that those with more severe FSHD have lower parasympathetic output and higher sympathetic output, compared to less‐affected FSHD patients. In addition, lower HRV has been observed in patients suffering from Duchenne and Becker muscular dystrophy (Ammendola et al., 2006; Lanza et al., 2001; Yotsukura et al., 1995). This dysfunction has been described as an increase in sympathetic output and a decrease in parasympathetic output as compared with healthy controls (Lanza et al., 2001; Yotsukura et al., 1995). While decreased HF power and increased LF power was observed in the muscular dystrophy group of these studies, it is important to note that LF power is not a marker of sympathetic output and has considerable influence from parasympathetic activity and the baroreflex (Goldstein et al., 2011; Randall et al., 1976). Thus, results from these previous studies should be considered with caution.

Notably, Tsuji and colleagues observed a significant association between decreased HF power and cardiovascular mortality in older adults (Tsuji et al., 1994, 1996). Since decreased parasympathetic drive has the potential to increase an individual's risk of developing CVD, these findings may indicate increased risk for cardiac events in individuals with FSHD (Grassi, 2009; Thayer et al., 2010). Our findings, however, are not consistent with these previous studies. In the current study, FSHD patients exhibited similar HF power, RMSSD and SDNN to their control counterparts. While both sympathetic and parasympathetic activity contribute to SDNN, HF power and RMSSD are vagally mediated and indicate that parasympathetic function is similar between the two groups in our study (Shaffer & Ginsberg, 2017). Besides one FSHD participant taking a prescribed anti‐hypertensive medication, our FSHD cohort was relatively healthy without cardiac dysfunction, which could be the reason BRS was not attenuated in adults with FSHD in this study. The lack of attenuation in BRS and HRV in adults with FSHD supports the notion that autonomic function is likely spared in adults with FSHD, without existing co‐morbidities.

### 4.3 Physical deconditioning in FSHD

Physical deconditioning as a result of limited mobility and physical activity can lead to numerous health‐related outcomes such as negative changes in body composition, decreased sensitivity of blood pressure regulation, as well as the occurrence of dysglycemia and dyslipidemia (Maher et al., 2017), contributing to an elevated CVD risk profile (Nash et al., 2005). In the present study, the FSHD group exhibited lower activity levels than the control group with an AMI of 24 ± 30 kcal/day (FSHD) compared to 222 ± 175 kcal/day (CTL). Vera et al. observed that individuals with FSHD experience exercise intolerance (Vera et al., 2022), likely a contributing factor to deconditioning in this group. Further, several studies have reported that BRS decreases following periods of physical deconditioning (Convertino et al., 1990; Convertino & Fritsch, 1992), although these studies focused on prolonged bed rest rather than disease‐related limited mobility.

Elevated resting HR can be a marker of sympathetic overdrive and is commonly associated with increased risk of CVD and cardiac events (Zhang et al., 2016). Albeit in normal ranges, we observed an elevated HR in adults with FSHD as compared with controls which was no longer significant when co‐varying for physical activity levels. Collectively, these results suggest an intact autonomic regulation of BP in this cohort of FSHD, and the observed elevated HR could likely be mitigated with increasing physical activity levels in adults with FSHD.

### 4.4 Limitations

It is important to consider limitations when interpreting results of the current study. First, the sample size was small, with a considerably lower number of female participants, precluding any sex‐difference analysis. Of note, however, effect sizes were also considerably low, indicating that regardless of sample size, the difference in means was minimal. Menstrual cycles were also not controlled for in the study, as two participants were post‐menopausal and one pre‐menopausal. While BRS has been reported to fluctuate throughout the menstrual cycle, we do not believe this severely impacted our results, due to the small sample of female participants (Brooks et al., 2012). Further, a third of the individuals in each group were on medications that can influence BRS (Broadley et al., 2005; Vasudev et al., 2011; Watkins & Grossman, 1999) which could have influenced the results of this study. However, when co‐varied for medications, the outcome of the analysis did not change. It is also important to note that a similar number of participants in each group were taking these prescribed medications. The study participants were also primarily European American, making the results less generalizable to other races and ethnicities.

## 5 CONCLUSION

The current study provides evidence that BRS and HRV in individuals with FSHD are preserved, despite the lower physical ability indicated by the FSHD‐HI. Further, our observations suggest that a slightly elevated HR in FSHD may be indicative of physical inactivity or deconditioning, which may put this group at risk for future CVD or cardiac events, but this could be secondary to FSHD or overexpression of the DUX4 gene mutation, as we did not observe alterations in autonomic function in this group. Clinically, these findings are important because they may suggest that the greater CVD that previous studies have observed could be linked to deconditioning in addition to the misregulation of the DUX4 gene. With this knowledge, we are in a stronger position to suggest therapeutic interventions that would be beneficial to reduce CVD risk in FSHD. For example, it has been demonstrated that exercise can lead to significant functional benefits without compromising muscle tissue in patients with FSHD (Bankolé et al., 2016). Therefore, patients with FSHD may benefit from increasing physical activity levels both to optimize functional status and reduce the risk of CVD. Future research should explore specific exercise recommendations for the FSHD population and their influence on the cardiovascular risk profile in these individuals.

## CONFLICT OF INTEREST

The authors of this research have no disclosures or competing interests.

## ETHICAL APPROVAL

All study procedures were approved by the University of Minnesota Institutional Review Board and conducted in accordance with the Declaration of Helsinki.

## AUTHOR CONTRIBUTIONS

MK‐R and MK conceived the presented study. MK‐R, KV, MM, and SC participated in data collection; data organization was completed by MM, KV, ML, MA, EL, and SC. ML and MA primarily completed data analysis. MA, SC, and EL were primarily responsible for manuscript development. Editorial guidance was provided by MK‐R.

## References

[phy215277-bib-0001] Ammendola, E. , Russo, V. , Politano, L. , Santangelo, L. , & Calabrò, R. (2006). Is heart rate variability a valid parameter to predict sudden death in patients with Becker’s muscular dystrophy? Heart, 92(11), 1686–1687. 10.1136/hrt.2005.082909 17041120 PMC1861247

[phy215277-bib-0002] Bankolé, L.‐C. , Millet, G. Y. , Temesi, J. , Bachasson, D. , Ravelojaona, M. , Wuyam, B. , Verges, S. , Ponsot, E. , Antoine, J.‐C. , Kadi, F. , & Féasson, L. (2016). Safety and efficacy of a 6‐month home‐based exercise program in patients with facioscapulohumeral muscular dystrophy: A randomized controlled trial. Medicine, 95(31), e4497. 10.1097/MD.0000000000004497 27495097 PMC4979851

[phy215277-bib-0003] Berlit, P. , & Stegaru‐Hellring, B. (1991). The heart in muscular dystrophy: An electrocardiographic and ultrasound study of 20 patients. European Archives of Psychiatry and Clinical Neuroscience, 241(3), 177–180. 10.1007/BF02219718 1790164

[phy215277-bib-0004] Bertinieri, G. , di Rienzo, M. , Cavallazzi, A. , Ferrari, A. U. , Pedotti, A. , & Mancia, G. (1985). A new approach to analysis of the arterial baroreflex. Journal of Hypertension. Supplement, 3(3), S79–S81. 2856787

[phy215277-bib-0005] Billman, G. E. , Schwartz, P. J. , & Stone, H. L. (1982). Baroreceptor reflex control of heart rate: A predictor of sudden cardiac death. Circulation, 66(4), 874–880. 10.1161/01.CIR.66.4.874 7116603

[phy215277-bib-0006] Bosnakovski, D. , Xu, Z. , Gang, E. J. , Galindo, C. L. , Liu, M. , Simsek, T. , Garner, H. R. , Agha‐Mohammadi, S. , Tassin, A. , Coppée, F. , Belayew, A. , Perlingeiro, R. R. , & Kyba, M. (2008). An isogenetic myoblast expression screen identifies DUX4‐mediated FSHD‐associated molecular pathologies. EMBO Journal, 27(20), 2766–2779. 10.1038/emboj.2008.201 PMC2572182 18833193

[phy215277-bib-0007] Broadley, A. J. M. , Frenneaux, M. P. , Moskvina, V. , Jones, C. J. H. , & Korszun, A. (2005). Baroreflex sensitivity is reduced in depression. Psychosomatic Medicine, 67(4), 648–651. 10.1097/01.psy.0000170829.91643.24 16046382

[phy215277-bib-0008] Brooks, V. L. , Cassaglia, P. A. , Zhao, D. , & Goldman, R. K. (2012). Baroreflex function in females: Changes with the reproductive cycle and pregnancy. Gender Medicine, 9(2), 61–67. 10.1016/j.genm.2012.02.004 22483197 PMC3350105

[phy215277-bib-0009] Chu, V. , Otero, J. M. , Lopez, O. , Sullivan, M. F. , Morgan, J. P. , Amende, I. , & Hampton, T. G. (2002). Electrocardiographic findings in mdx mice: A cardiac phenotype of Duchenne muscular dystrophy. Muscle and Nerve, 26(4), 513–519. 10.1002/mus.10223 12362417

[phy215277-bib-0010] Convertino, V. A. , Doerr, D. F. , Eckberg, D. L. , Fritsch, J. M. , & Vernikos‐Danellis, J. (1990). Head‐down bed rest impairs vagal baroreflex responses and provokes orthostatic hypotension. Journal of Applied Physiology, 68(4), 1458–1464. 10.1152/jappl.1990.68.4.1458 2347788

[phy215277-bib-0011] Convertino, V. A. , & Fritsch, J. M. (1992). Attenuation of human carotid‐cardiac vagal baroreflex responses after physical detraining. Aviation, Space and Environmental Medicine, 63(9), 785–788. 1524534

[phy215277-bib-0012] Coupé, M. , Fortrat, J. O. , Larina, I. , Gauquelin‐Koch, G. , Gharib, C. , & Custaud, M. A. (2009). Cardiovascular deconditioning: From autonomic nervous system to microvascular dysfunctions. Respiratory Physiology & Neurobiology, 169(Suppl 1), S10–S12. 10.1016/j.resp.2009.04.009 19379845

[phy215277-bib-0013] Cygankiewicz, I. , & Zareba, W. (2013). Heart rate variability. Handbook of Clinical Neurology, 117, 379–393. 24095141 10.1016/B978-0-444-53491-0.00031-6

[phy215277-bib-0014] Della Marca, G. , Frusciante, R. , Scatena, M. , Dittoni, S. , Testani, E. , Vollono, C. , Losurdo, A. , Scarano, E. , Colicchio, S. , Farina, B. , & Gnoni, V. (2010). Heart rate variability in facioscapulohumeral muscular dystrophy. Functional Neurology, 25(4), 211–216. 21388582

[phy215277-bib-0015] Dewey, K. G. (1997). Energy and protein requirements during lactation. Annual Review of Nutrition, 17, 19–36. 10.1146/annurev.nutr.17.1.19 9240917

[phy215277-bib-0016] Ducharme‐Smith, A. , Nicolau, S. , Chahal, C. A. A. , Ducharme‐Smith, K. , Rehman, S. , Jaliparthy, K. , Khan, N. , Scott, C. G. , St Louis, E. K. , Liewluck, T. , Somers, V. K. , Lin, G. , Brady, P. A. , & Milone, M. (2021). Cardiac involvement in Facioscapulohumeral Muscular Dystrophy (FSHD). Front Neurol, 12, 668180. 10.3389/fneur.2021.668180 34108930 PMC8181417

[phy215277-bib-0017] Emmrich, P. , Ogunlade, V. , Gradistanac, T. , Daneschnejad, S. , Koch, M. C. , & Schober, R. (2005). Facioscapulohumeral muscle dystrophy and heart disease. Zeitschrift Fur Kardiologie, 94(5), 348–354. 15868364 10.1007/s00392-005-0223-4

[phy215277-bib-0018] Finsterer, J. , Stöllberger, C. , & Meng, G. (2005). Cardiac involvement in facioscapulohumeral muscular dystrophy. Cardiology, 103(2), 81–83. 10.1159/000082113 15550754

[phy215277-bib-0019] Geng, L. N. , Yao, Z. , Snider, L. , Fong, A. P. , Cech, J. N. , Young, J. M. , van der Maarel, S. M. , Ruzzo, W. L. , Gentleman, R. C. , Tawil, R. , & Tapscott, S. J. (2012). DUX4 activates germline genes, retroelements, and immune mediators: Implications for facioscapulohumeral dystrophy. Developmental Cell, 22(1), 38–51. 10.1016/j.devcel.2011.11.013 22209328 PMC3264808

[phy215277-bib-0020] Goldstein, D. S. , Bentho, O. , Park, M.‐Y. , & Sharabi, Y. (2011). Low‐frequency power of heart rate variability is not a measure of cardiac sympathetic tone but may be a measure of modulation of cardiac autonomic outflows by baroreflexes. Experimental Physiology, 96(12), 1255–1261. 10.1113/expphysiol.2010.056259 21890520 PMC3224799

[phy215277-bib-0021] Gordin, D. , Vikatmaa, P. , Vikatmaa, L. , Groop, P.‐H. , Albäck, A. , & Tikkanen, I. (2016). Baroreflex activation therapy in the treatment of resistant hypertension. Duodecim, 132(20), 1874–1881. 29190040

[phy215277-bib-0022] Grassi, G. (2009). Assessment of sympathetic cardiovascular drive in human hypertension: Achievements and perspectives. Hypertension, 54(4), 690–697. 10.1161/HYPERTENSIONAHA.108.119883 19720958

[phy215277-bib-0023] Hoaglin, D. C. , Iglewicz, B. , & Tukey, J. W. (1986). Performance of some resistant rules for outlier labeling. Journal of the American Statistical Association. 81(396), 991–999. 10.1080/01621459.1986.10478363

[phy215277-bib-0024] Hughson, R. L. , & Shoemaker, J. K. (2015). Autonomic responses to exercise: Deconditioning/inactivity. Autonomic Neuroscience, 188, 32–35. 10.1016/j.autneu.2014.10.012 25458429

[phy215277-bib-0025] Johnson, N. E. , Quinn, C. , Eastwood, E. , Tawil, R. , & Heatwole, C. R. (2012). Patient‐identified disease burden in facioscapulohumeral muscular dystrophy. Muscle and Nerve, 46(6), 951–953. 10.1002/mus.23529 23225386 PMC4097080

[phy215277-bib-0026] La Rovere, M. T. , Pinna, G. D. , Hohnloser, S. H. , Marcus, F. I. , Mortara, A. , Nohara, R. , Bigger, J. T. , Camm, A. J. , & Schwartz, P. J. (2001). Baroreflex sensitivity and heart rate variability in the identification of patients at risk for life‐threatening arrhythmias: Implications for clinical trials. Circulation, 103(16), 2072–2077. 10.1161/01.CIR.103.16.2072 11319197

[phy215277-bib-0027] Laforêt, P. , de Toma, C. , Eymard, B. , Becane, H. M. , Jeanpierre, M. , Fardeau, M. & Duboc, D. (1998). Cardiac involvement in genetically confirmed facioscapulohumeral muscular dystrophy. Neurology, 51(5), 1454–1456. 10.1212/WNL.51.5.1454 9818880

[phy215277-bib-0028] Lanfranchi, P. A. , & Somers, V. K. (2002). Arterial baroreflex function and cardiovascular variability: Interactions and implications. American Journal of Physiology: Regulatory, Integrative and Comparative Physiology, 283(4), R815–R826. 10.1152/ajpregu.00051.2002 12228049

[phy215277-bib-0029] Lanza, G. A. , Dello Russo, A. , Giglio, V. , De Luca, L. , Messano, L. , Santini, C. , Ricci, E. , Damiani, A. , Fumagalli, G. , De Martino, G. , Mangiola, F. , & Bellocci, F. (2001). Impairment of cardiac autonomic function in patients with Duchenne muscular dystrophy: Relationship to myocardial and respiratory function. American Heart Journal, 141(5), 808–812. 10.1067/mhj.2001.114804 11320370

[phy215277-bib-0030] Lim, K. R. Q. , Nguyen, Q. , & Yokota, T. (2020). DUX4 signalling in the pathogenesis of facioscapulohumeral muscular dystrophy. International Journal of Molecular Sciences, 21(3), 729. 10.3390/ijms21030729 PMC7037115 31979100

[phy215277-bib-0031] Lof, M. , Olausson, H. , Bostrom, K. , Janerot‐Sjöberg, B. , Sohlstrom, A. , & Forsum, E. (2005). Changes in basal metabolic rate during pregnancy in relation to changes in body weight and composition, cardiac output, insulin‐like growth factor I, and thyroid hormones and in relation to fetal growth. American Journal of Clinical Nutrition, 81(3), 678–685. 10.1093/ajcn/81.3.678 15755839

[phy215277-bib-0032] Lohmeier, T. E. (2001). The sympathetic nervous system and long‐term blood pressure regulation. American Journal of Hypertension, 14(6), 147S–154S. 10.1016/S0895-7061(01)02082-9 11411750

[phy215277-bib-0033] Lohmeier, T. E. , Irwin, E. D. , Rossing, M. A. , Serdar, D. J. , & Kieval, R. S. (2004). Prolonged activation of the baroreflex produces sustained hypotension. Hypertension, 43(2), 306–311. 10.1161/01.HYP.0000111837.73693.9b 14707159

[phy215277-bib-0034] Lucini, D. , Mela, G. S. , Malliani, A. , & Pagani, M. (2002). Impairment in cardiac autonomic regulation preceding arterial hypertension in humans: Insights from spectral analysis of beat‐by‐beat cardiovascular variability. Circulation, 106(21), 2673–2679. 10.1161/01.CIR.0000039106.89299.AB 12438292

[phy215277-bib-0035] Maher, J. L. , McMillan, D. W. , & Nash, M. S. (2017). Exercise and health‐related risks of physical deconditioning after spinal cord injury. Topics in Spinal Cord Injury Rehabilitation, 23(3), 175–187. 10.1310/sci2303-175 29339894 PMC5562026

[phy215277-bib-0036] Martinka, P. , Fielitz, J. , Patzak, A. , Regitz‐Zagrosek, V. , Persson, P. B. , & Stauss, H. M. (2005). Mechanisms of blood pressure variability‐induced cardiac hypertrophy and dysfunction in mice with impaired baroreflex. American Journal of Physiology: Regulatory, Integrative and Comparative Physiology, 288(3), R767–R776. 10.1152/ajpregu.00445.2004 15563577

[phy215277-bib-0037] Mavrogeni, S. I. , Markousis‐Mavrogenis, G. , Papavasiliou, A. , Papadopoulos, G. , & Kolovou, G. (2018). Cardiac involvement in duchenne muscular dystrophy and related dystrophinopathies. In C. Bernardini (Ed.), Duchenne muscular dystrophy: Methods and protocols (pp. 31–42). New York, NY. 10.1007/978-1-4939-7374-3_3 29067654

[phy215277-bib-0038] Nash, M. S. , DeGroot, J. , Martinez‐Arizala, A. , & Mendez, A. J. (2005). Evidence for an exaggerated postprandial lipemia in chronic paraplegia. Journal of Spinal Cord Medicine, 28(4), 320–325. 10.1080/10790268.2005.11753827 PMC1864900 16396382

[phy215277-bib-0039] Ormezzano, O. , Cracowski, J.‐L. , Quesada, J.‐L. , Pierre, H. , Mallion, J.‐M. , & Baguet, J.‐P. (2008). EVAluation of the prognostic value of BARoreflex sensitivity in hypertensive patients: The EVABAR study. Journal of Hypertension, 26(7), 1373–1378. 10.1097/HJH.0b013e3283015e5a 18551013

[phy215277-bib-0040] Parati, G. , Di Rienzo, M. , & Mancia, G. (2000). How to measure baroreflex sensitivity: From the cardiovascular laboratory to daily life. Journal of Hypertension, 18(1), 7–19. 10.1097/00004872-200018010-00003 10678538

[phy215277-bib-0041] Pomeranz, B. , Macaulay, R. J. , Caudill, M. A. , Kutz, I. , Adam, D. , Gordon, D. , Kilborn, K. M. , Barger, A. C. , Shannon, D. C. , & Cohen, R. J. (1985). Assessment of autonomic function in humans by heart rate spectral analysis. American Journal of Physiology, 248(1), H151–H153. 10.1152/ajpheart.1985.248.1.H151 3970172

[phy215277-bib-0042] Preston, M. K. , Tawil, R. , & Wang, L. H. (1999). Facioscapulohumeral muscular dystrophy. In M. P. Adam , H. H. Ardinger , R. A. Pagon , S. E. Wallace , L. J. H. Bean , K. W. Gripp , G. M. Mirzaa , & A. Amemiya (Eds.), GeneReviews®. University of Washington, Seattle. 20301616

[phy215277-bib-0043] Randall, D. C. , Brown, D. R. , Raisch, R. M. , Yingling, J. D. , & Randall, W. C. (1991). SA nodal parasympathectomy delineates autonomic control of heart rate power spectrum. American Journal of Physiology, 260(3), H985–H988. 10.1152/ajpheart.1991.260.3.H985 1672056

[phy215277-bib-0044] Randall, O. S. , Esler, M. D. , Bulloch, E. G. , Maisel, A. S. , Ellis, C. N. , Zweifler, A. J. & Julius, S. (1976). Relationship of age and blood pressure to baroreflex sensitivity and arterial compliance in man. Clinical Science, 3, 357s–360s. 10.1042/cs051357s 1071645

[phy215277-bib-0045] Richardson, M. T. , Leon, A. S. , Jacobs, D. R. Jr , Ainsworth, B. E. , & Serfass, R. (1994). Comprehensive evaluation of the minnesota leisure time physical activity questionnaire. Journal of Clinical Epidemiology, 47(3), 271–281. 10.1016/0895-4356(94)90008-6 8138837

[phy215277-bib-0046] Rudas, L. , Crossman, A. A. , Morillo, C. A. , Halliwill, J. R. , Tahvanainen, K. U. , Kuusela, T. A. & Eckberg, D. L. (1999). Human sympathetic and vagal baroreflex responses to sequential nitroprusside and phenylephrine. American Journal of Physiology, 276(5), H1691–H1698. 10.1152/ajpheart.1999.276.5.H1691 10330255

[phy215277-bib-0047] Sabharwal, R. , Weiss, R. M. , Zimmerman, K. , Domenig, O. , Cicha, M. Z. , & Chapleau, M. W. (2015). Angiotensin‐dependent autonomic dysregulation precedes dilated cardiomyopathy in a mouse model of muscular dystrophy. Experimental Physiology, 100(7), 776–795. 10.1113/EP085066 25921929 PMC4505616

[phy215277-bib-0048] Shaffer, F. , & Ginsberg, J. P. (2017). An Overview of heart rate variability metrics and norms. Frontiers in Public Health, 5, 258. 10.3389/fpubh.2017.00258 29034226 PMC5624990

[phy215277-bib-0049] Smyth, H. S. , Sleight, P. , & Pickering, G. W. (1969). Reflex regulation of arterial pressure during sleep in man. A quantitative method of assessing baroreflex sensitivity. Circulation Research, 24(1), 109–121. 10.1161/01.RES.24.1.109 4303309

[phy215277-bib-0050] Statland, J. M. , & Tawil, R. (2016). Facioscapulohumeral Muscular Dystrophy . Continuum. 22(6, Muscle and Neuromuscular Junction Disorders):1916–31. 10.1212/CON.0000000000000399 PMC5898965 27922500

[phy215277-bib-0051] Stevenson, W. G. , Perloff, J. K. , Weiss, J. N. , & Anderson, T. L. (1990). Facioscapulohumeral muscular dystrophy: Evidence for selective, genetic electrophysiologic cardiac involvement. Journal of the American College of Cardiology, 15(2), 292–299. 10.1016/S0735-1097(10)80052-X 2299071

[phy215277-bib-0052] Tawil, R. , Kissel, J. T. , Heatwole, C. , Pandya, S. , Gronseth, G. , & Benatar, M. (2015). Evidence‐based guideline summary: Evaluation, diagnosis, and management of facioscapulohumeral muscular dystrophy: Report of the guideline development, dissemination, and implementation subcommittee of the American Academy of Neurology and the Practice Issues Review Panel of the American Association of Neuromuscular & Electrodiagnostic Medicine. Neurology, 85(4), 357–364. 10.1212/WNL.0000000000001783 26215877 PMC4520817

[phy215277-bib-0053] Thames, M. D. , Kinugawa, T. , Smith, M. L. , & Dibner‐Dunlap, M. E. (1993). Abnormalities of baroreflex control in heart failure. Journal of the American College of Cardiology. 22(4), A56–A60. 10.1016/0735-1097(93)90464-C 8104206

[phy215277-bib-0054] Thayer, J. F. , Yamamoto, S. S. , & Brosschot, J. F. (2010). The relationship of autonomic imbalance, heart rate variability and cardiovascular disease risk factors. International Journal of Cardiology, 141(2), 122–131. 10.1016/j.ijcard.2009.09.543 19910061

[phy215277-bib-0055] Trevisan, C. P. , Pastorello, E. , Armani, M. , Angelini, C. , Nante, G. , Tomelleri, G. , Tonin, P. , Mongini, T. , Palmucci, L. , Galluzzi, G. , Tupler, R. G. , & Barchitta, A. (2006). Facioscapulohumeral muscular dystrophy and occurrence of heart arrhythmia. European Neurology, 56(1), 1–5. 10.1159/000094248 16804309

[phy215277-bib-0056] Tsuji, H. , Larson, M. G. , Venditti, F. J. Jr , Manders, E. S. , Evans, J. C. , Feldman, C. L. & Levy, D. (1996). Impact of reduced heart rate variability on risk for cardiac events. The Framingham Heart Study. Circulation, 94(11), 2850–2855. 10.1161/01.CIR.94.11.2850 8941112

[phy215277-bib-0057] Tsuji, H. , Venditti, F. J. Jr , Manders, E. S. , Evans, J. C. , Larson, M. G. , Feldman, C. L. , & Levy, D. (1994). Reduced heart rate variability and mortality risk in an elderly cohort. The Framingham Heart Study. Circulation, 90(2), 878–883. 10.1161/01.CIR.90.2.878 8044959

[phy215277-bib-0058] Vasudev, A. , O’Brien, J. T. , Tan, M. P. , Parry, S. W. , & Thomas, A. J. (2011). A study of orthostatic hypotension, heart rate variability and baroreflex sensitivity in late‐life depression. Journal of Affective Disorders, 131(1–3), 374–378. 10.1016/j.jad.2010.11.001 21122918

[phy215277-bib-0059] Vera, K. , McConville, M. , Glazos, A. , Stokes, W. , Kyba, M. , & Keller‐Ross, M. (2022). Exercise intolerance in facioscapulohumeral muscular dystrophy. Medicine & Science in Sports & Exercise. Published Ahead of Print. Available from: 10.1249/MSS.0000000000002882 PMC9117420 35195100

[phy215277-bib-0060] Vera, K. A. , McConville, M. , Kyba, M. , & Keller‐Ross, M. L. (2020). Sarcopenic obesity in facioscapulohumeral muscular dystrophy. Frontiers in Physiology, 11, 1008. 10.3389/fphys.2020.01008 32903446 PMC7435048

[phy215277-bib-0061] Vera, K. , McConville, M. , Kyba, M. , & Keller‐Ross, M. (2021). Resting metabolic rate in adults with facioscapulohumeral muscular dystrophy. Applied Physiology, Nutrition and Metabolism, 46(9), 1058–1064. 10.1139/apnm-2020-1119 33735584

[phy215277-bib-0062] Wang, L. H. , & Tawil, R. (2016). Facioscapulohumeral dystrophy. Current Neurology and Neuroscience Reports, 16(7), 66. 10.1007/s11910-016-0667-0 27215221

[phy215277-bib-0063] Watkins, L. L. , Fainman, C. , Dimsdale, J. , & Ziegler, M. G. (1995). Assessment of baroreflex control from beat‐to‐beat blood pressure and heart rate changes: A validation study. Psychophysiology, 32(4), 411–414. 10.1111/j.1469-8986.1995.tb01224.x 7652118

[phy215277-bib-0064] Watkins, L. L. , & Grossman, P. (1999). Association of depressive symptoms with reduced baroreflex cardiac control in coronary artery disease. American Heart Journal, 137(3), 453–457. 10.1016/S0002-8703(99)70491-6 10047625

[phy215277-bib-0065] Wessel, N. , Gapelyuk, A. , Kraemer, J. F. , Berg, K. , & Kurths, J. (2020). Spontaneous baroreflex sensitivity: Sequence method at rest does not quantify causal interactions but rather determines the heart rate to blood pressure variability ratio. Physiological Measurement. 10.1088/1361-6579/ab7edc 32160607

[phy215277-bib-0066] Yotsukura, M. , Sasaki, K. , Kachi, E. , Sasaki, A. , Ishihara, T. , & Ishikawa, K. (1995). Circadian rhythm and variability of heart rate in Duchenne‐type progressive muscular dystrophy. American Journal of Cardiology, 76(12), 947–951. 10.1016/S0002-9149(99)80267-7 7484837

[phy215277-bib-0067] Zhang, D. , Wang, W. , & Li, F. (2016). Association between resting heart rate and coronary artery disease, stroke, sudden death and noncardiovascular diseases: A meta‐analysis. Canadian Medical Association Journal, 188(15), E384–E392. 10.1503/cmaj.160050 27551034 PMC5056889

